# Supplementary material for: Computational characterization of the xanthan gum glycosyltransferase GumK
Source: PLoS Comput Biol. 2025 Dec 10;21(12):e1013803. doi: 10.1371/journal.pcbi.1013803 (PMC12768416; doi:10.1371/journal.pcbi.1013803)
Supplement: S1 Table — (DOCX) [file pcbi.1013803.s022.docx]

| **Region** | **Residue** | **Function** |
| --- | --- | --- |
| Donor domain | Lys307, Tyr292, Ser305, Ser230, Arg29 | Sugar diphosphate interaction |
|  | Leu301, Met231, Leu232, Met306 | Steric constrain |
| Acceptor domain | Leu56, Val89, Phe92, Lys60, Arg96 | Tail interaction |
|  | Glu131, Ser22, Ser132, His23, Arg52 | Sugar interaction |
| Interdomain | Arg29, Glu272 | Opening |
|  | Tyr328, His201, Ser160, Glu192, Asp157, Thr161, Asp303, Ser186, Met189, Ser304 | Twisting |
