# Supplementary material for: Computational characterization of the xanthan gum glycosyltransferase GumK
Source: PLoS Comput Biol. 2025 Dec 10;21(12):e1013803. doi: 10.1371/journal.pcbi.1013803 (PMC12768416; doi:10.1371/journal.pcbi.1013803)
Supplement: Attachment — Submitted filename: Rebuttal_Letter_Luciano2025_2.pdf [file pcbi.1013803.s023.pdf]

Dear Prof. Chen,

Please find enclosed the revised version of the manuscript PCOMPBIOL-D-25-01831 with the title “Computational characterization of the xanthan gum glycosyltransferase GumK”.

We were very pleased by the positive comments and the constructive suggestions provided by thereviewers. As you will see from a detailed description provided below, we have been able to handle their comments in a straightforward and non-controversial manner. The revisions primarily address methodological clarifications, including the rationale for force field selection, validation of the ClustENMD conformational ensemble, and addition of a comprehensive summary table (Table 1) of all simulations performed. We have also included several new supplementary figures to support these points.

To facilitate reviewing the revision, we attach versions of the revised manuscript in which changes are marked using *latexdiff*.

We hope that our revised manuscript is acceptable for publication, and we look forward to your response.

Sincerely,

Gaston Courtade

Reviewer #1

The authors reported their computer simulation findings for GumK, a glycosyltransferase from Xanthomonas campestris (a Gram negative bacteria) required for xanthan gum biosynthesis. The authors performed a series of normal mode analyses, CG metadynamics, all-atom MD, Hamiltoniam REMD, and bioinformatics sequence analyses to provide potential mechanistic insight and conformational dynamics behavior for GumK. Given the industry application and its pathogenicity to plants, the study is of general scientific interest. The authors provided a systematic study with incremental complexity in modeling and simulating the putative mode of membrane/substrate binding, and protein dynamics to elucidate its enzymatic function. There are some major concerns about the simulated systems I would like the authors to address prior to further consideration for publication.

We thank the reviewer for their positive comments about the scientific relevance of our work. We address the concerns raised below.

Why did the authors use a less accurate, predicted AlphaFold structure as a starting point for CG metadynamics MD simulation when a high resolution of X-ray structures available (resolution for unbound GumK is 1.9 angstrom)? Did the author perform structure comparison analysis between the predicted and X-ray structure. While AlphaFold is well suited in predicting the 3D fold of the structure based on AI training from previously solved structure, it cannot predict structures on the unsolved N and C terminus of GumK and has problems in predicting the sidechain packing of the protein.

The AlphaFold-predicted structure was carefully compared with the available X-ray crystal structure (PDB ID: 2HY7). The backbone RMSD between the two models is approximately 0.4 Å, and 0.5 Å for just the side chains, indicating a high degree of structural similarity. We clarified this point by adding the following sentence “The predicted structure shows an RMSD of the backbone, with respect to the crystal structure, of 0.4Å and an RMSD of 0.5Å when including the side chains in the structural alignment” in the results section “Charged residues in the acceptor domain mediate interactions between GumK and the membrane”. Fig. S7 has been added to show this comparison. The main motivation for using the AlphaFold model was that it provides a reasonable starting point for the unresolved N- and C-terminal regions, which are missing in the crystallographic structure. Importantly, in the coarse-grained metadynamics simulations, these terminal regions were not constrained by the elastic network and were therefore allowed to rearrange and interact freely with the membrane environment. The only structural bias introduced by the MARTINI force field concerns the maintenance of the assigned secondary structure elements. In other GT70 family members, AlphaFold

predicts the terminal regions as partially helical. In our simulations, we deliberately avoided imposing this helical bias and focused instead on evaluating the general interaction pattern of the terminal regions with the membrane.

How did the authors “docked” the GumK to the membrane? From Figure 3B, it seems a portion of the N-terminal domain was inserted into the membrane. What was the rationale behind it? Was it based primarily on Fig 5 illustration from ref 12?

We employed two complementary approaches to evaluate how GumK interacts with the membrane as stated in results section “Charged residues in the acceptor domain mediate interactions 326 between GumK and the membrane” lines 333-349. First, we used the OPM webserver, which estimates the optimal orientation of membrane proteins based on physical principles and hydrophobic transfer energies. Second, we performed coarse-grained OPES-metadynamics simulations using a modified MARTINI force field with rescaled protein–protein interactions which can better describe membrane-protein complexes compared to other corrections to MARTINI3 as shown in Thomasen et al. (https://doi.org/10.1038/s41467-024-50647-9). The rationale for the latter approach was to explore multiple possible membrane-binding modes without imposing a predefined docking orientation. Unlike OPM, which treats the protein as a rigid body and provides only one predicted pose, the OPES simulations allowed GumK to freely sample different orientations relative to the membrane. Importantly, the simulations were initiated from an unbound state, and the final bound configuration emerged spontaneously during the biased exploration. A cylindrical funnel restraint was applied solely to prevent unproductive diffusion of the protein into the bulk solvent, thus improving computational efficiency. Therefore, the observed docking mode in our simulations is not a result of artificial constraints but rather reflects the protein’s intrinsic tendency to associate with the membrane under the given conditions.

While ref 12 stated that GumK is a membrane-associated enzyme, and provided a putative mode of binding to the membrane, the region shown is highly hydrophilic with large number of positively charged residues, making membrane insertion highly unlikely (Fig 3). Was that the reason why the authors had to rescaled the protein-protein interaction and apply cylindrical-shaped restraining potential to maintain a stable docked complex within the membrane? As stated by the authors, the OPES “simulation is not fully converged” after 5 us of simulation. That is a major concern. What happens when these constraints were removed? Did the CaRMSD of the -terminal region collapse, or did it flew out of the membrane?

We thank the reviewer for this observation, which allows us to clarify an important aspect of our methodology. The protein was not artificially maintained in a docked conformation. The final pose emerged spontaneously during the OPES-metadynamics simulations, in which GumK was free to explore multiple orientations with respect to the membrane surface. The resulting bound state corresponds to a minimum on the computed free-energy surface, indicating that it represents a stable and energetically favorable configuration rather than an artifact of the restraints. In this minimum, the protein penetrates the membrane through residues Trp98 and Trp109, which insert into the lipid monolayer and act as anchoring points. Tryptophan residues have been reported in the literature as canonical membrane-anchoring motifs in monotopic enzymes (refs. 36 newly added and 47), supporting the physiological relevance of the observed binding mode. The overall stability of the docked configuration arises from a combination of hydrophobic interactions at the phospholipid interface and electrostatic stabilization involving positively charged residues that interact with the negatively charged phospholipid headgroups. This mixed interaction pattern is consistent with the behavior expected for monotopic integral membrane proteins such as GumK. The cylindrical funnel restraint applied during the simulations served exclusively to prevent unproductive diffusion of the protein into the bulk solvent, thereby improving sampling efficiency, but it did not constrain either the orientation or the depth of membrane insertion. When this restraint was removed in control simulations, the protein remained stably associated with the membrane. Finally, although the OPES simulations were not fully converged, the free-energy shows a clear minimum corresponding to the observed docked pose, indicating that the system had attained a representative and physically meaningful bound configuration. To make the aim clearer, we made the following changes in methods: “We used an OPES simulation to study how GumK interacts with the membrane” and “The starting conformation is placed under the membrane, and it is in an unbound state”. Rephrased the following sentence in Results: “The elastic network in MARTINI constrained the protein conformation to the closed one”

Membrane-associated does not imply membrane-inserted. There is a lack of strong support for the proposed membrane bound mechanism in ref 12 Fig 5. Given the acceptor substrate (Man-Cel-UNDDP) consists of a long unsaturated aliphatic chain, it is more likely the membrane-association of GumK is due to its binding to the membrane inserted acceptor substrate. This would allow the cationic site in the N-terminal domain be placed directly above the zwitterionic headgroup of the membrane lipids for favorable electrostatic complementary interactions.

We agree that membrane association does not necessarily imply membrane insertion. However, based on the literature and our simulation results discussed above, we support

the interpretation that GumK behaves as a monotopic integral membrane protein and that our simulations capture its most probable membrane orientation. A comparative example can be found in PimA, a peripheral glycosyltransferase that contains several charged residues at the membrane interface but lacks the anchoring tryptophans (such as Trp98 and Trp109 in GumK) typically found in monotopic integral proteins. This difference likely explains why PimA remains peripheral, while GumK exhibits a stable partial insertion into the membrane.

Please provide a figure of the acceptor-protein complex used for all atomistic unbiased MD simulation. How was the acceptor substrate docked into the protein-membrane complex. Was the simulation done with both donor and acceptor substrate. It was not clear from the method section. Also, was the complex system model based on the endpoint from the meta dynamics simulation? The bound structure of GumK (PDB: 2Q6V) only has the donor substrate? Modeling of the acceptor substrate into glycosyl transferase has been a challenge to many previous simulation studies.

The starting complex for the all-atom unbiased MD simulations was constructed manually by positioning the acceptor substrate near the region where a phospholipid molecule transiently bound in the unbiased simulation of the free protein as stated in lines 557-558 of Results section "The acceptor substrate is constrained in a linear conformation by the binding site". This site was therefore identified as a likely acceptor-binding region. During the subsequent simulation, the hydrophobic tail of the acceptor inserted spontaneously into the lipid monolayer, while the glycan portion established transient contacts with the protein surface. Due to the high flexibility and complexity of the acceptor, a stably bound conformation was observed in only one out of three independent replicas started from the same initial model. The protein conformation used for these simulations was derived from the OPM-predicted orientation, which is directly compatible with CHARMM-GUI, facilitating system preparation. We selected this structure because it closely matches the membrane-bound pose obtained from the OPES-metadynamics simulations, while excluding the terminal regions that are not directly involved in substrate recognition. We note that the available crystal structure (PDB 2Q6V) contains only the nucleoside moiety of the donor substrate, which does not provide sufficient information for reliable modeling of the entire donor–acceptor–protein complex. For this reason, and to avoid introducing artificial constraints on the catalytic site, we focused on the interaction between GumK and the acceptor substrate alone, which already presented a significant modeling challenge, as also pointed out by the reviewer. A representative figure of the acceptor–protein complex used as the starting point for the unbiased MD simulation has now been included in the revised Supporting Information (Fig. S18).

Why did the authors perform NPT instead of the typical NPAT simulation for membranes?

The use of the NPT ensemble in this case was not driven by a specific methodological requirement but rather by continuity with preliminary simulations of the protein in solution, which were performed under NPT conditions (not reported in the manuscript). Nevertheless, we are aware that NPAT conditions are often preferred for membrane systems, and we therefore carefully verified the numerical stability of the simulation. The system density and area per lipid remained stable throughout the trajectory, showing only minor fluctuations and no signs of anomalous compression or instability typically associated with inappropriate barostat settings. These data are reported in the figure below, confirming that the membrane maintained its physical integrity under the applied NPT conditions.

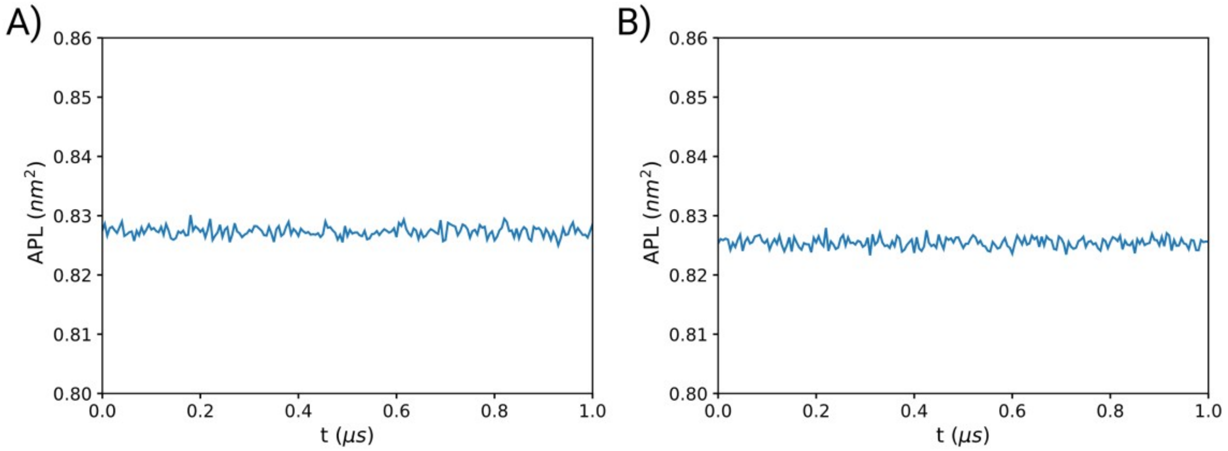


Area per lipid (APL) during the unbiased MD simulations. (A) GumK in the absence of the acceptor substrate. (B) GumK interacting with the acceptor substrate. In both cases, the APL remains constant with a low standard deviation and shows no drift over time, indicating that the membrane is stable and well equilibrated.

Previous studies have shown unbound and “closed” glycosyltransferase can undergo open-close conformations during MD simulation. It is essential for the N and C terminal domains of the unbound enzyme to "open" for substrate binding. The rationale behind using "closed" conformation avoids sampling interdomain dynamics may be flawed.

The closed conformation of GumK was maintained by the Martini elastic network, as the coarse-grained force field does not accurately reproduce protein–protein (interdomain) interactions required for realistic open–close transitions. Consequently, restraining the domains in their closed arrangement prevents unphysical unfolding and ensures structural stability throughout the simulation. The goal of these simulations was not to investigate the interdomain dynamics, but rather to characterize how the protein interacts with the membrane. Based on experimental and structural data, the N-terminal domain is hypothesized to mediate initial contact with the membrane. Allowing the protein to undergo domain opening in this coarse-grained framework would have introduced unrealistic flexibility and confounded the interpretation of membrane-binding behavior.

It is the “opened” conformation that enable the modeling of the bound substrate into glycosyltransferase. The potential that restrained the unbound “closed” conformation would not favor the sampling of the bound modes, as stated by the authors.

We agree that the open conformation is essential for substrate accommodation. Importantly, in the unbiased all-atom simulations, the protein was not constrained to remain in the closed state. In these simulations, GumK remained associated with the membrane both in the presence and absence of the acceptor substrate, and it exhibited a natural tendency to partially open due to the intrinsic chemistry of the interdomain interface. When the acceptor substrate was included, the protein was also free to move, and the substrate spontaneously bound within the amphiphilic cleft between the two domains. This interaction further promoted a modest opening motion, consistent with a conformational response upon substrate binding. Overall, our all-atom simulations suggest that GumK remains tablybound to the membrane without interacting with any substrates and undergo domain opening thanks to the acceptor interaction.

Please confirm that the restraints were removed in the production run in the unbiased MD simulation.

We added te following sentence “In all unbiased MD simulations, no constraints were applied to GumK, allowing the protein to undergo conformational changes freely.” in the method section “Full atomistic unbiased MD simulation”.

Reviewer #2

This manuscript provides a very comprehensive computational study of the GT-B glycosyltransferase GumK, a key enzyme in xanthan gum biosynthesis. The authors employ multi-scale simulation approaches (NMA, ClustENMD, coarse-grained and all-atom MD, HREX) combined with bioinformatic analysis to probe the enzyme's conformational dynamics, membrane interaction, and substrate specificity. The work addresses an important and underexplored area in glycosyltransferase biology. The findings on the substrate-dependent catalytic cycle (acceptor binding promotes opening, donor binding stabilizes closure) and the identification of key residues for specificity are novel and significant. However, the manuscript requires revision to improve before it can be recommended for publication.

We thank the reviewer for their positive comments about the scientific relevance of our work. We address the concerns raised in the following.

The authors utilize different force fields (CHARMM36m for unbiased MD/HREX, AMBER99SB-ILDN in ClustENMD). A clear justification for this choice is absent. The reader is left wondering if this was due to technical constraints of the different software packages or a conscious choice based on the specific strengths of each force field for the different types of simulations. The authors should briefly explain this strategy in the Methods section to preempt concerns about consistency.

We thank the reviewer for pointing out this ambiguity. To adress this we have added the sentence "energy-minimized using the default force field in ClustENMD, AMBER99SB-ILDN" in the method section "Normal mode analysis and ClustENMD". A second sentence in the method section "Full atomistic unbiased MD simulation" was added to clarify the use of CHARMM36: "This choice was motivated by the convenient use of CHARMM-GUI for building protein–membrane systems, and because CHARMM36m provides consistent parameters for both proteins and lipids. In contrast, the AMBER99SB-ILDN force field implemented in ClustENMD is primarily optimized for soluble proteins"

The conformational ensemble from ClustENMD (Fig. 2) is a key starting point for the study. Are these conformations stable, or are they transient states sampled due to the specific constraints of the ClustENMD method? The authors should strengthen their argument by: Explicitly stating whether any of the major conformational states (especially the open states) were observed and were stable in the subsequent, more physiologically relevant, unbiased MD simulations of the membrane-bound protein (the 1 μs trajectory). If not observed in the unbiased MD, the authors should discuss the limitations of the

ClustENMD approach in the context of a membrane-anchored protein and temper their conclusions accordingly.

We appreciate the reviewer raising this important point about the physical relevance of the ClustENMD ensemble. To address this concern, we have added the following paragraph to the discussion section 'The interdomain contacts can tune GumK activity':

> It is important to note that ClustENMD may generate unphysical conformations characterized by distorted secondary structures and/or overstretched loops, due to the external forces applied along the deformation vectors [48]. Not all structures sampled by ClustENMD necessarily belong to the ensemble of conformations populated in solution. The applied force tends to deform the secondary structure of the domains rather than promoting a genuine opening, leading to unphysical distortions. To quantify the extent of these deformations, we compared each domain to the starting structure using RMSD. In the acceptor domain, 8.9% of the conformations exceed an RMSD of 2 Å, while in the donor domain this fraction is 3.1%. Furthermore, a subset of the conformations sampled by ClustENMD is also observed in the fully atomistic MD simulations (Fig. S10), supporting the reliability of ClustENMD as a method to generate open conformations of GT-B glycosyltransferases.

The manuscript employs a large number of simulations with different system setups (protein alone, protein+membrane, protein+donor, protein+acceptor, etc.). A table summarizing all simulations performed would be immensely helpful. This table should include: Simulation Type (e.g., Unbiased MD, HREX), System Description (e.g., Full GumK + membrane, Donor Domain + GlcA-UDP), Force Field, Simulation Time, and Key Objective. This would greatly enhance the readability and allow readers to easily grasp the full scope of the computational work.

This is an excellent point. A new Table 1 has been added in the Methods section, summarizing all the simulations performed, together with a short introductory paragraph to facilitate the understanding of the different system setups and their objectives
